# Crude preparation of a phage-encoded biofilm-dispersing factor expressed in *E. coli* and its potential application in bacteriological analysis of environmental water samples

**DOI:** 10.1128/aem.01163-25

**Published:** 2025-10-16

**Authors:** Shah Nayeem Faruque, Iftekhar Bin Naser, M. Mozammel Hoque, Fariya Akter, Shah M. Faruque

**Affiliations:** 1 Laboratory Sciences and Services Division, International Centre for Diarrhoeal Disease Research, Bangladesh 46647 , Dhaka, Bangladesh; 2 Department of Mathematics and Natural Sciences, BRAC University 118864, Dhaka, Bangladesh; 3 School of Environment and Life Sciences, Independent University Bangladesh, Dhaka, Bangladesh; University of Nebraska-Lincoln, Lincoln, Nebraska, USA

**Keywords:** biofilms, *Vibrio cholerae*, bacteriophage, biofilm-dispersing factor

## Abstract

A fundamental technical challenge in detecting pathogenic bacteria in aquatic reservoirs is the inability to accurately estimate biofilm-associated cells in water. Considering the role of biofilms in environmental persistence and waterborne transmission of bacterial pathogens, there is an increasing interest in substances that can effectively degrade bacterial biofilms. The biofilm-dispersing *Vibrio cholerae* phage JSF7 was analyzed by whole genome sequencing and found to carry a gene predicted to encode an enzyme for degrading complex polysaccharides. The gene was cloned in *Escherichia coli* DH5α*,* and crude extract from the recombinant *E. coli* enhanced the dispersion of diverse bacterial biofilms, including those of *E. coli*, *Shigella dysenteriae*, *Pseudomonas aeruginosa*, and *V. cholerae*. The crude extract was fully active at a temperature of 37°C and pH of 7.0 but was inactivated by proteinase-K treatment. Analysis of environmental water samples for the presence of *V. cholerae* O1 by enrichment culture detected significantly more *V. cholerae* O1-positive samples when the enrichment medium was supplemented with the extract, as compared with typical enrichment without the extract. These results suggest that a crude preparation of the phage-encoded biofilm-degrading factor expressed in *E. coli* has potential application in degrading bacterial biofilms and enhancing bacteriological analysis of water.

**IMPORTANCE:**

In their aquatic reservoirs, bacteria often exist as biofilms and are difficult to accurately detect by culturing water samples. Such biofilms have been implicated in waterborne transmission of pathogenic bacteria. We identified a bacteriophage that can disintegrate biofilms and disperse biofilm-associated bacteria. The putative phage gene responsible for this activity was cloned in an *E. coli* strain, and the crude cellular extract of the recombinant *E. coli* was found to promote dispersion of a variety of bacterial biofilms. Supplementation of bacterial growth medium with the crude extract also enhanced detection of *V. cholerae* O1, the causative agent of cholera in environmental water samples. The ability of a phage-derived biofilm-degrading factor to disperse diverse bacterial biofilms provides a novel approach for enhancing detection of waterborne bacterial pathogens in water beyond traditional enrichment methods.

## INTRODUCTION

Bacterial biofilms are a significant source of infection and microbial contamination in medical and industrial settings, as well as in the enhanced waterborne transmission of pathogens (1–3). Biofilms are communities of bacteria adhering to surfaces and are embedded in a complex matrix of extracellular polymeric substances often composed of polysaccharides, proteins, nucleic acids, and lipids (1–4). Pathogenic bacteria in biofilms often show enhanced resistance to antimicrobials (5, 6). These bacteria are thus difficult to eliminate and can become a persistent source of the pathogen.

In the aquatic environment, pathogenic bacteria often exist in biofilms, and their transmission is facilitated by the aggregated structure, which can deliver a high dose of the pathogen to a potential host (3, 7). For example, toxigenic *Vibrio cholerae,* which can cause epidemics of cholera, exist predominantly as biofilms in aquatic reservoirs and infect humans (3, 8). In light of observations that suggest that biofilms are an important source of persistent infections and waterborne transmission of bacterial pathogens, as well as a means of evading antimicrobial treatment (1–6), there is an increasing interest in enzymes that can degrade bacterial biofilms. Such enzymes are also likely to have application in improving detection of bacterial pathogens in environmental water samples, by dispersion of biofilm-associated bacteria. We previously identified a *V. cholerae* O1-specific bacteriophage designated as JSF7, which can act on biofilms and disperse biofilm-associated bacterial cells (9). In the present study, we cloned and expressed the JSF7 phage-encoded biofilm-dispersing factor in *Escherichia coli* and demonstrated that a crude extract from the recombinant clone can be used in degrading diverse bacterial biofilms, as well as in enhancing microbiological analysis of environmental water samples to detect pathogenic *V. cholerae*.

## MATERIALS AND METHODS

### Culture of phage and bacterial strains

Bacterial strains included in this study for phage propagation and biofilm assays were obtained from our culture collection at icddr,b. Description of microbial strains, plasmids, genetic constructs, and the phage used in this study are presented in Table 1. Luria-Bertani broth (LB) or LB-agar plates were routinely used to culture various bacterial strains. Whenever applicable, selective media, including McConkey agar, tellarite-taurocholate gelatin agar (TTGA), or *Pseudomonas* agar plates were used to selectively grow *E. coli*, *V. cholerae*, and *P. aeruginosa,* respectively. The biofilm-degrading phage JSF7 was initially isolated from a sample of environmental water in Bangladesh as described previously (9). For routinely amplifying the phage, a *V. cholerae* O1 host strain C6706 was cultured overnight for 16 h in LB, diluted 1:100 in fresh LB, and grown at 37°C for 4 h. A sample of the phage obtained from a single plaque was added to the bacterial culture and incubated at 37°C for 16 h. Supernatant of the culture obtained after centrifugation for 20 min at 10,000*×g* was made bacteria-free by filtration using 0.22-µm pore size filter (Merc Millipore Ltd., Cork, Ireland). The number of phage particles in the filtered supernatant was determined by the soft agar overlay method as described previously (9, 10).

**TABLE 1:** Description of bacterial strains, phage, and plasmids included in this study

Strain/plasmid	Description	Reference
JSF7	*Vibrio cholerae* O1-specific bacteriophage isolated from samples of environmental water in Bangladesh	(9)
C6706	*V. cholerae* O1 El Tor clinical isolate	(7)
AI1852	*V. cholerae* O139 clinical strain	Laboratory collection
PA-2123516	*Pseudomonas aeruginosa* clinical strain	Laboratory collection
SD-33891	*Shigella dysenteriae* Type 1 clinical strain	Laboratory collection
pNSF18	PCR amplified ORF30 cloned in pGEM vector	This study
DH5α (pGEM)	*E. coli* DH5α carrying empty pGEM cloning vector	This study
DH5α (pNSF18)	*E. coli* DH5α carrying pNSF18	This study

### Isolation of JSF7 phage DNA

Phage particles were precipitated from the filter-sterilized culture supernatants by centrifuging a mixture of the supernatant with one-fourth volume of PEG-NaCl solution (20% polyethylene glycol, PEG-6000, and 10% NaCl). The phage pellet was resuspended in a buffer containing 20 mM Tris-Cl (pH 7.5), 10 mM MgCl, 60 mM KCl, and 10 mM NaCl. To remove exogenous DNA and RNA, the solution was treated for 2 h at 37°C with DNAse I (100 units/mL) and RNAse A (50 µg/mL) (Promega Corporation, Madison, USA). Phage particles were then disrupted by extraction with phenol-chloroform, and the DNA was precipitated by using ethanol. The DNA was dissolved in deionized water and further purified using the Promega DNA purification system (SV Minipreps; Promega).

### Analysis of phage genome

Illumina-based technology was used for sequencing of the phage genome as described previously (9). Briefly, libraries were prepared using Illumina Nextera XT DNA library Preparation Kit as per instructions of the manufacturer. The Illumina MiSeq system was used for sequencing, and online software available at Illumina BaseSpace was used to assemble sequences into contigs and aligned with reference sequences. Genomic sequence of JSF7 phage has been deposited to GenBank and is available under GenBank accession number KY065149.

### Cloning of phage gene in *E. coli*

**Fig 1:**
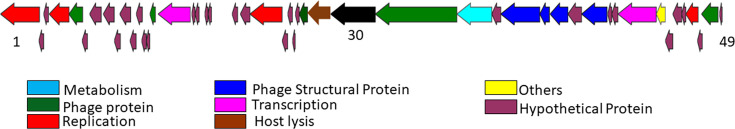
Genome map of the JSF7 phage showing open reading frames (ORFs) with their size and transcriptional orientation. ORF30, predicted to encode a polysaccharide-degrading enzyme in JSF7, is marked in black.

The JSF7 phage gene predicted to encode a putative biofilm-degrading factor was cloned using standard molecular methods (11). Briefly, ORF30 of the JSF phage genome (Fig. 1) encoding a putative polysaccharide-degrading enzyme was amplified using PCR with the primers GCGTCGGCTTTAAGTGTTGT and GAAGCAACAGGTGCGGTTAT. The amplicon was cloned using pGEM-T Easy Vector Systems (Promega) to construct pNSF18, which was introduced into competent *E. coli* DH5α cells. Recombinants were selected by blue/white screening after growing for 16 h at 37°C on LB agar plates containing ampicillin (50 µg/mL), 0.1 mM isopropyl β-D-1-thiogalactopyranoside (IPTG), and X-Gal (50 µg/mL). Suspected recombinant colonies were picked and further analyzed to confirm the presence of the recombinant plasmid. Subsequently, total protein extracts of *E. coli* DH5α (pNSF18) and *E. coli* DH5α carrying the empty vector were analyzed by sodium dodecyl sulfate polyacrylamide gel electrophoresis (SDS-PAGE) to observe possible expression of any new protein in the recombinant strain as described below.

### SDS-PAGE analysis

Transformed *E. coli* DH5α containing pNSF18 or the empty vector was grown in LB with shaking at 37°C until the cell density reached an OD_600_ of 0.8. IPTG was added to the cultures at a final concentration of 1 mM, and incubation was continued at 37°C for 12 h. Then, 1 mL of the culture was centrifuged for 5 min at 10,000 × *g* to precipitate cells. The cell pellet was re-suspended in 250 µL of distilled water, and an equal volume of 2× SDS sample buffer (20% glycerol, 4% SDS, 100 mM Tris-Cl pH 6.8; 0.001% bromophenol blue and 0.05% β-mercaptoethanol). The suspension was heated at 95°C for 10 min, mixed by vortexing, and centrifuged to separate insoluble cellular debris from soluble proteins. Aliquots of the supernatant containing soluble proteins were loaded into a 12% SDS-polyacrylamide gel, and electrophoresis was conducted. Protein bands were visualized by staining with Coomassie Brilliant Blue R-250 solution (Bio-Rad, CA, USA).

### Preparation of crude cellular extracts

Bacterial cells were grown in LB with IPTG as described above, and 15 mL of the culture was centrifuged for 5 min at 10,000 × *g* to collect cell pellets. The cells were lysed on ice using a lysis buffer composed of 50 mM Tris (pH 7.4), 300 mM NaCl, 10 µM leupeptin, 1 mM phenylmethylsulfonyl fluoride (PMSF), aprotinin (2 µg/mL), and pepstatin (10 µg/mL). Cell lysates were dialyzed for 12 h in 10 mM Tris-Cl, pH 7.4 at 4°C, changing the dialysis buffer every 2 h for five times. The dialyzed extract was stocked as a crude preparation of the biofilm-degrading factor. Serial dilutions of this extract were initially tested for biofilm-degrading activity using biofilms of *V. cholerae* strain C6706 as described below. *V. cholerae* C6706 biofilms were dispersed by approximately 50% (observed reduction in absorbance at 570 nm) in 3 h, when a 10-fold dilution of the stock was used. Subsequently, a 10-fold dilution of the stock was used as the working dilution for all assays.

### Preparation and estimation of biofilms

Appropriate bacterial strains were used to form biofilms on the inner surfaces of borosilicate glass tubes using previously described methods (12). Briefly, bacterial colonies grown on LB-agar plates were picked and suspended in LB broth to an optical density of 0.6 at 600 nm. Multiple borosilicate glass tubes were inoculated with aliquots of the bacterial suspension after diluting 100 folds in LB, and these cultures were allowed to stand at room temperature for 36 h to form biofilms. The biofilms were estimated using the crystal violet staining method in which tubes containing biofilms were rinsed with distilled water to remove non-adherent cells, and then filled with 1 mL of a 0.1% crystal violet solution (Sigma-Aldrich, Inc., St. Louis, USA). After 30 min, the tubes containing biofilms were again rinsed with water to remove non-adherent dye. One milliliter of dimethyl sulfoxide (DMSO) was added to extract the biofilm-associated crystal violet dye in the tubes. For estimation of biofilms, the optical density of the resulting suspension was read at 570 nm, and the reading was interpreted as described previously (12).

### Assay for biofilm-dispersing activity

To estimate biofilm-dispersing capability of the cellular extract of *E. coli* DH5α (pNSF18), biofilm prepared as described above was exposed to the extract and examined for degradation of biofilms and increase in cell count due to release of free cells. Appropriate strains were used to produce biofilms in a series of glass tubes as described above. Free cells were washed away, and tubes with biofilm attached to the inner surfaces were retained. The biofilm contained in the tubes was incubated with 1 mL of the 10× diluted stock extract in PBS (pH 7.0) and incubated at 37°C. Control tubes were incubated in parallel with 1 mL of lysate prepared from *E. coli* cells carrying the empty vector. At different time intervals, planktonic cell counts in the aqueous phase were determined. Biofilm retained in the tubes after exposure to the extract was also estimated as described above.

### Effect of pH, temperature, and proteinase-K treatment

To examine the effect of pH and temperature on the activity of the biofilm-dispersing factor, aliquots (0.5 mL) of the extract prepared as described above were inoculated into series of tubes containing 4.5 mL of PBS adjusted to a defined pH and analyzed for activity on biofilms of *V. cholerae* strain C6706 as described above. Initially, activity was examined at three different pH (6.0, 7.0, and 8.0) and temperature (25°C, 37°C, and 50°C) and at combinations of these parameters. Later, a more elaborate examination of the effect of pH (3, 5, 7, 9, 11, and 13) at 37°C was done. The effect of temperature (25°C, 30°C, 37°C, 45°C, 50°C, and 55°C) on the activity of the factor was examined at pH 7.0. Overall, the activity was found to be highest at pH 7.0 and at a temperature range of 35°C to 40°C. Subsequently, for all other assays, the temperature and pH were 37°C and 7.0, respectively.

To examine the effect of proteinase-K, 0.5 mL of the cellular extract was mixed with 0.5 mL buffer (20 mM Tris-Cl pH 8.0, 50 mM EDTA, 1% SDS). Proteinase K (Cat no. V3021; 36 u/mg Promega) was added to a final concentration of 50 µg/mL, and the reaction was incubated at 37°C for 2 h. The activity of the proteinase-K treated extract was then tested on biofilms of *V. cholerae* strain C6706 as described above.

### Isolation of *V. cholerae* O1 from water samples

Samples of environmental water selected for this assay were initially found negative for the presence of *V. cholerae* O1 in conventional enrichment cultures. Dialyzed cellular extract (500 µL) of *E. coli* DH5α (pNSF18) was added to 2.0 mL of water along with 2.5 mL of 5× concentrated bile peptone medium (BP, 1% peptone, 0.5% taurocholic acid, 1% NaCl, pH 9.0), and was incubated at 37°C for 5 h. Samples enriched with extract of *E. coli* DH5α containing the empty vector were also incubated in parallel as control assays. Dilutions of the enrichment cultures were plated on taurocholate tellurite gelatin agar (TTGA) containing streptomycin (70 µg/mL) following the AST enrichment culture technique (13). Individual colonies of suspected *V. cholerae* O1 were picked and subjected to standard serological tests for confirmation and for the virulence genes *ctxA* and *tcpA* using specific PCR assays as described previously (13).

### Statistical analysis

The statistical analysis program built-in Microsoft Excel (version 2016) was used to calculate mean ± standard deviation when appropriate, and the differences were tested by two-tailed *t*-test. Values of *P* < 0.05 were considered statistically significant.

## RESULTS

### JSF7 phage genome carries the gene for a putative biofilm-degrading factor

Analysis of the complete genomic sequence of JSF7 phage revealed a total of 49 ORFs within the genome comprising 46,310 base pairs (Fig. 1). Almost 39% of the predicted ORFs (19 of 49 ORFs) of the JSF7 phage were found to have predictive functions (Fig. 1). These included ORFs encoding six DNA metabolism-related proteins, including DNA polymerase, DNA helicase, phage DNA packaging, DNA ligase, phage RNA polymerase. Five ORFs were predicted to encode specific phage-related proteins. There were four ORFs that code for phage structural proteins including phage capsid protein, phage tail fiber protein, tail protein, and phage collar protein. Remarkably, the genome of JSF7 carried an ORF (designated as ORF 30; 2,796 bp), which was predicted to encode a polysaccharide-degrading enzyme, pectin lyase/cycloalternan hydrolase and was suspected to be the biofilm-degrading factor. As described above, this ORF was cloned and expressed in *E. coli* DH5α to confirm biofilm degrading activity of the cellular extract of the recombinant strain. The genomic sequence of JSF7 phage and the sequence of the putative biofilm-degrading factor is available under GenBank accession number KY065149.

### Extract of *E. coli* carrying cloned JSF7 phage ORF30 disperses bacterial biofilms

The ORF30, which was predicted to encode a polysaccharide degrading enzyme, was amplified by PCR from JSF7 phage DNA and cloned into pGEM-T cloning vector to construct pNSF18 and introduced into *E. coli* DH5α. Analysis of total protein extracted from *E. coli* DH5α (pNSF18) and *E. coli* DH5α carrying the empty vector by SDS-PAGE showed an additional protein band of ~37 kDa in the extract of *E. coli* DH5α (pNSF18) but not in that of *E. coli* DH5α carrying the empty vector (data not shown).

**Fig 2:**
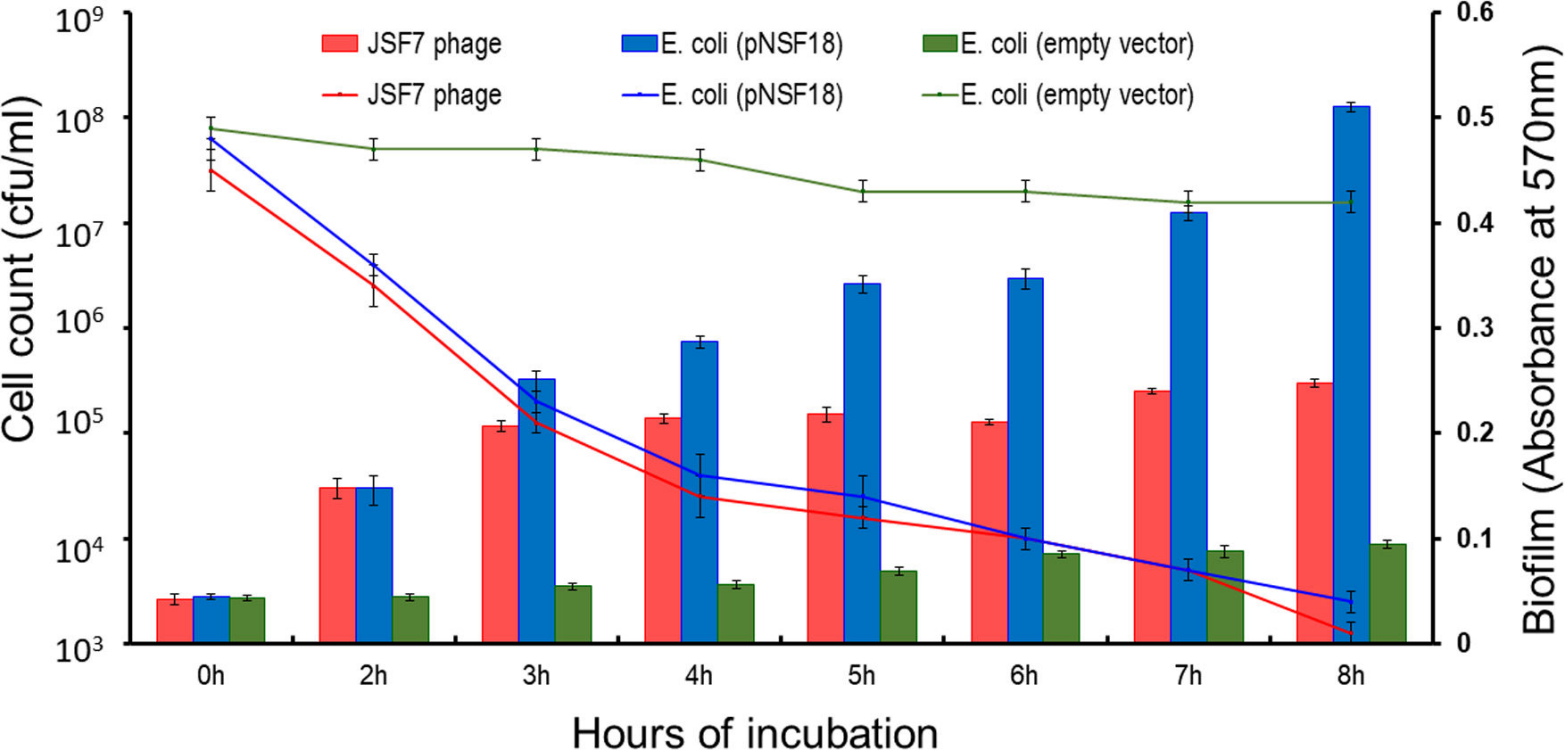
Biofilm dispersion of *V. cholerae* O1 strain C6706 by intact JSF7 phage or cloned biofilm-degrading factor, with corresponding increase in planktonic cells (bar graph). The increase in planktonic cell count with intact phage was lower than treatment with cloned product, since the strain was susceptible to the phage.

**Fig 3:**
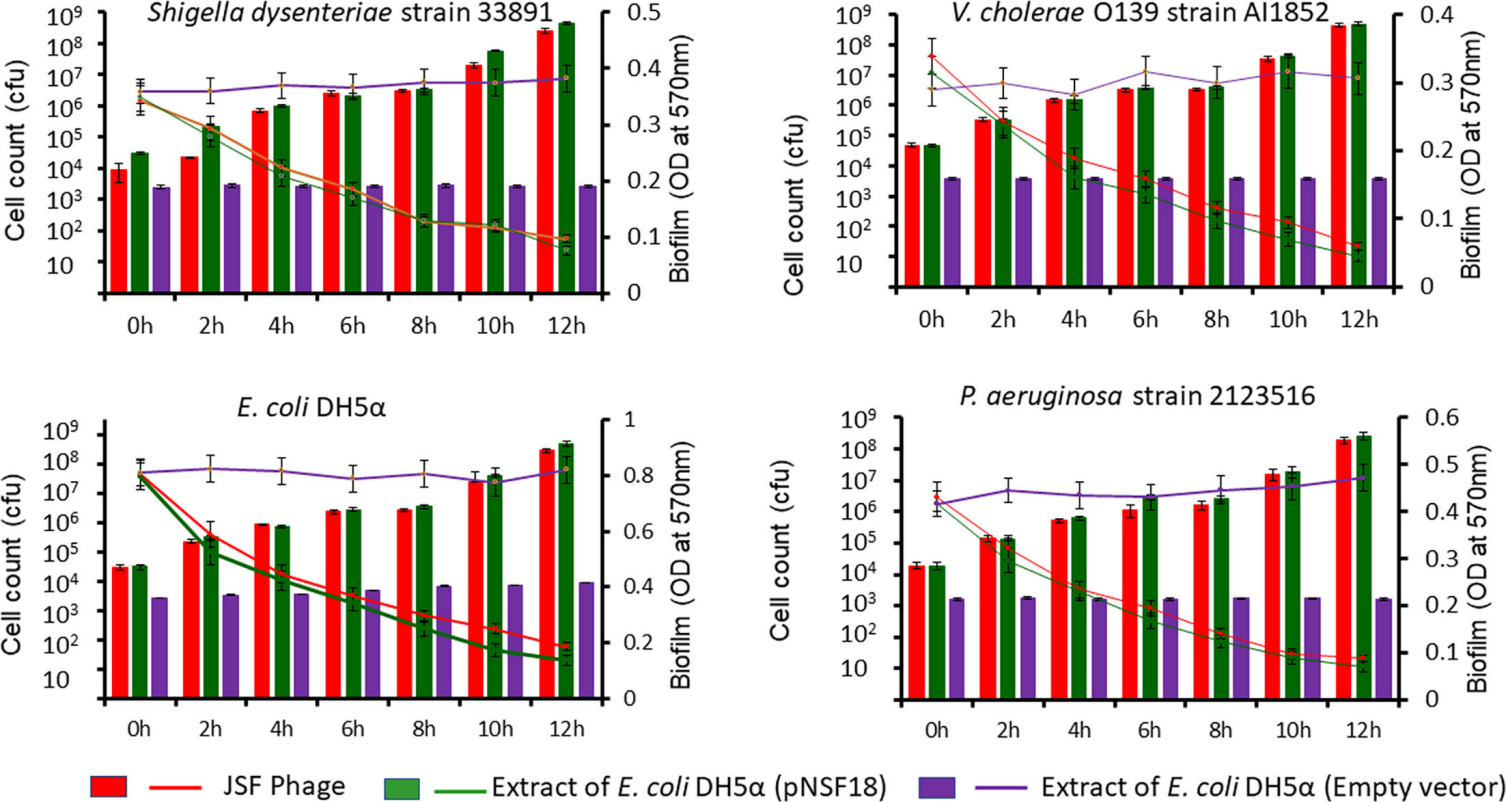
Biofilm dispersion of diverse bacterial strains by intact JSF7 phage or cloned biofilm-degrading factor and resulting increase in planktonic cell count (bar graphs). Purple lines and bars show the result of similar experiments using extract of *E. coli* DH5α carrying the empty vector.

Extract of cell lysate of the recombinant clone encoding the putative polysaccharide degrading factor and intact JSF7 phage were compared for biofilm-degrading activity using biofilms produced by *V. cholerae* strain C6706 (Fig. 2). Exposure to either the intact JSF7 phage or the extract of the recombinant clone led to dispersion of biofilm-associated cells, as was evident from a decrease in biofilm and a concomitant increase in bacterial cell count. Similarly, biofilm-degrading activity of the cellular extract of *E. coli* (pNSF18) was observed on monomicrobial biofilms of *E. coli* DH5α, *P. aeruginosa,* and *S. dysenteriae* 1 strains included in this study (Fig. 3).

### The JSF7-ORF30 gene product enhances detection of *V. cholerae* O1 in water samples.

Since the crude preparation containing the cloned biofilm-degrading factor was found to decompose biofilms under laboratory conditions, we examined whether this preparation also enhanced recovery of possible biofilm-associated bacteria in water samples collected in environmental sites in Bangladesh. To exclude samples that might contain a few cells of planktonic *V. cholerae* O1 cells, aliquots of water were tested by enrichment culture for *V. cholerae* as described previously (7), and only samples found to be negative in this initial screening were included in this assay. We found that the addition of the extract from *E. coli* DH5α carrying the cloned ORF30 (50% vol:vol) significantly increased the detection of *V. cholerae* O1 in many of the water samples after enrichment for a few hours (Table 2). When enriched and cultured in an identical manner but exposed to extract from control cultures of *E. coli* DH5α carrying the empty cloning vector, aliquots of these same water samples were mostly found to be negative for *V. cholerae* O1 (Table 2).

**TABLE 2:** Effect of cloned JSF7-ORF30 gene product on recovery of *V. cholerae* O1 from surface water in Bangladesh

Site of sampling	No. of samples tested	No. of samples found positive for *V. cholerae* O1 after enrichment in LB containing cellular extract of various strains^*a*^		
		*E. coli* DH5α	*E. coli* DH5α (pGEM)	*E. coli* DH5α (pNSF18)
Turag River	12	2	1	8
Buriganga River	12	1	1	8
Gulshan Lake	12	1	2	7
Mohakhali Lake	12	0	0	9
Gabtoli swamp	12	0	1	7
Total	60	4	5	39
% of total	100	6.66	8.33	65.0

^*a*^ LB broth medium supplemented with 50% (v/v) cellular extract of the indicated strain were mixed with the samples, and incubated for 6 h at 37°C. Aliquots of the enrichment culture were plated on TTGA plates containing streptomycin. The observed differences in isolation of *V. cholerae* O1 when enriched in the presence of cellular extract of *E. coli* DH5α (pNSF18) were statistically significant (*P* < 0.001) when compared with that of either of the assays using extract of *E. coli* DH5α or *E. coli* DH5α containing the empty vector. Only surface water samples found negative by routine enrichment culture were included in this assay and shown in the table.

## DISCUSSION

Bacteria and bacteriophages are ubiquitous in the aquatic environment and are important components of the overall ecological settings. While bacteria in the environment often persist as biofilms, occasionally phages possess biofilm-degrading enzymes to be able to access biofilm-associated bacteria that are assumed to remain otherwise unaffected by phages (9). JSF7 represents such a phage that can prey on biofilm-associated *V. cholerae* O1 and was originally isolated from the aquatic environment in Bangladesh, where cholera is endemic. Of 36 different *V. cholerae*-specific phages isolated from water (9), only the JSF7 phage was capable of degrading biofilms. On analysis of the genomic sequence of JSF7 phage, the 2,796 bp ORF designated as ORF30 was predicted to encode an enzyme known as pectin lyase or pectinase (Fig. 1), which belongs to a group of enzymes referred to as glycoside hydrolases (14). In a previous study, pectinase from *Rhizopus* spp. was found to disperse mono-microbial and polymicrobial biofilms with high dispersal efficacy *in vitro* (14). The present study was designed to clone the putative JSF7 phage gene responsible for biofilm dispersion and express it in *E. coli* so that a crude cell extract of the recombinant strain could be conveniently prepared and applied in enhancing bacteriological analysis of environmental water samples, which often contain biofilms of bacteria that resist cultivation by usual methods. Thus, the crude cellular extract of *E. coli* DH5α carrying the recombinant plasmid pNSF18 was tested for degrading bacterial biofilms prepared in the laboratory, and in analysis of environmental water samples for *V. cholerae* O1, which often causes cholera in Bangladesh.

The bacterial strains used to prepare these monomicrobial biofilms included not only JSF7 phage-susceptible *V. cholerae* O1, such as strain C6706, but also other bacteria that are not susceptible to JSF7 phage, such as strains of *S. dysenteriae*, *P. aeruginosa*, and *V. cholerae* O139. The extract of recombinant *E. coli* DH5α (pNSF18) was able to degrade all these different biofilms tested (Fig. 3). When intact phage was used to degrade biofilms of *V. cholerae* O1 strain C6706, the concomitant increase in planktonic cell count with decrease in biofilms was comparatively less compared to that when extract of the recombinant clone was used (Fig. 2). This disparity was expected because *V. cholerae* O1 strain C6706 was susceptible to JSF7 phage, and so a part of the planktonic cells released from biofilms was supposedly lysed by the phage. On the contrary, degradation of biofilms of bacteria, such as *S. dysenteriae*, *P. aeruginosa*, or *V. cholerae* O139, led to a steady increase in planktonic cell count when either intact phage or the cloned gene product was used.

The cholera pathogen *V. cholerae* is known to persist in aquatic reservoirs in regions where cholera is endemic. However, isolating the pathogen from water samples is difficult because of their existence as biofilm-like aggregates of dormant cells known as conditionally viable environmental cells (CVECs) (3, 7, 15), which are difficult to grow by conventional enrichment and culture techniques. However, these latent cells are capable of being periodically resuscitated under natural conditions and cause outbreaks of cholera (15). Recovery of actively growing *V. cholerae* O1 by dispersion of CVEC has been demonstrated previously (16) using autoinducers which enhance dispersion of biofilms through the quorum sensing pathway (16, 17). Quorum sensing refers to a regulatory response in bacteria based on cell density that occurs through the sensing of extracellular autoinducers, which are produced by the bacterial community (17). In contrast, in the present study, we used a phage-encoded enzyme, which was cloned and expressed in *E. coli* to directly disperse biofilms and release planktonic cells of *V. cholerae* that could be detected by culture. These results suggest that the cloned gene product from JSF7 phage expressed in *E. coli* can be potentially used to enhance microbiological analysis of water samples. Notably, monitoring of microbiological quality of water constitutes a crucial step in following the transmission of waterborne diseases and predicting the risk of outbreaks.

The results of this study also provide several other important insights. Besides *V. cholerae*, many other pathogenic bacteria, including *V. vulnificus*, *Campylobacter jejuni*, *Shigella flexneri*, *Shigella sonnei,* and *S.* Enteritidis, have been found to assume the dormant state under adverse environmental conditions (18–20). In this study, we have shown that the cloned biofilm-degrading factor can act on biofilms of multiple species of bacteria (Fig. 3), suggesting that its application can be more widespread in analyzing environmental samples. In addition to *V. cholerae* O1 and *V. cholerae* O139, we demonstrated its activity on biofilms of other bacterial pathogens, such as *S. dysenteriae* 1 and *P. aeruginosa* (Fig. 3). These pathogenic bacteria were included in the assays to test the efficacy of biofilm degradation by the phage gene product, since eradication of biofilm-associated pathogens using antimicrobials is a challenge. Further studies will be directed towards investigating whether antimicrobial treatment of biofilm-associated bacterial infections in the presence of the phage-derived biofilm-degrading factor is more effective.

The present study also has several limitations that may be addressed in the future. Glycoside hydrolases (GHs) as a group include a number of enzymes such as α-amylase, alginate lyase, pectinase, amyloglucosidase, inulinase, and xylanase produced by different organisms and with different levels of biofilm-dispersing activity. In this study, ORF30 was predicted to encode a pectinase, and indeed, the recombinant product showed biofilm-degrading activity in the crude extract and was useful in enhancing bacteriological analysis of water. However, we did not purify the enzyme and did not study the biochemical parameters for the enzyme, which will be necessary before developing it for industrial or diagnostic applications. In a previous study (14), 16 GHs were screened for their ability to disperse biofilms grown in different environments, and certain differences were noted. Hence, a comparison of the biofilm-dispersing activity of the JSF7-derived enzyme with other available GHs would be useful. Similarly, there is a need to benchmark the effectiveness of the crude extract against other biofilm-dispersing strategies, such as commercial enzymes, chemical dispersants, or quorum sensing inducers (16). Finally, the observed enhanced detection of *V. cholerae* O1 in environmental water samples may be further evaluated with a larger number of samples from different environments.

### Conclusion

We identified a bacteriophage that can disintegrate bacterial biofilms and disperse biofilm-associated bacteria. We cloned the putative phage gene responsible for this activity in an *E. coli* strain and found that crude cellular extract of the recombinant *E. coli* could promote degradation of a variety of bacterial biofilms. Supplementation of bacterial growth medium with the crude extract also enhanced detection of *V. cholerae* O1 in environmental water samples. Thus, heterologous production of the phage-derived biofilm-degrading factor in *E. coli* has potential applicability in bacteriological analysis of water. The active factor was stable and active at temperatures around 37°C and pH 7.0, which makes it convenient to store and use it without maintaining a cold chain. However, the limitations of this study as described above emphasize the need for purification and further validation and comparison with existing methods of biofilm dispersion. Further purification and characterization of this biofilm-degrading factor might lead to its more efficient application, and future efforts will be directed towards this objective.
